# NATIONAL RECRUITMENT OF DIVERSE CAREGIVERS TO TEST AN ONLINE PLATFORM TO MANAGE BEHAVIORAL SYMPTOMS

**DOI:** 10.1093/geroni/igae098.1843

**Published:** 2024-12-31

**Authors:** Melinda Webster, Helen Kales, Lauren Parker, Daniel Scerpella, Katherine Marx, Nicole Bouranis, Laura Gitlin

**Affiliations:** Drexel University, Philadelphia, Pennsylvania, United States; University of California Davis, Davis, California, United States; Johns Hopkins Bloomberg School of Public Health, Baltimore, Maryland, United States; Johns Hopkins University, Baltimore, Maryland, United States; Johns Hopkins University, Baltimore, Maryland, United States; Yale University School of Medicine, New Haven, Connecticut, United States; Drexel University, Philadelphia, Pennsylvania, United States

## Abstract

Online platforms are promising scalable approaches to support diverse family caregivers. Testing these approaches requires recruiting nationally representative cohorts in terms of race, ethnicity, and geography. However, large-scale national recruitment is challenging given lack of best practices and inability to rely on local resources. We describe the recruitment sources used to enroll caregivers in an NIA-funded trial to test WeCareAdvisor, an online platform providing strategies to manage dementia-related behavioral symptoms. Over two years, 40 recruitment sources were pursued, yielding 892 inquiries from which 262 (29.3%) caregivers were enrolled. Recruitment sources were categorized into six types: 1) registries yielded the most study participants (550 inquiries, 112 enrolled, 42.7% of sample); 2) community-based providers/organizations (87 inquiries, 41 enrolled, 15.6%): 3) caregiver resources/respite services (50 inquiries, 34 enrolled, 13%); 4) dementia societies/associations/organizations (55 inquiries, 30 enrolled, 11.5%); 5) miscellaneous sources (87 inquiries, 23 enrolled, 8.8%); and 6) articles/professional talks (63 inquiries, 22 enrolled, 8.4%). Regarding diversity, registries yielded more non-spouses as compared to other recruitment sources; articles/professional talks yielded more racial/ethnic diversity proportionate to the number enrolled from that source (54.5% of 22 enrolled versus 15% of 112 enrolled through registries). Enrolled participants resided in 36 states, representing all 10 Department of Health & Human Services regions of the US. Costs (media ads, registry fees) totaled $22,438; $85.64 per participant. Although registries yielded the most enrollees, personal contact through articles/professional talks was more effective enrolling racially diverse caregivers. Recruiting caregivers nationally requires numerous strategies and fielding voluminous inquiries with implications for staffing/budgeting.

